# Association Between Sociodemographic Disparities and Door to Computerized Tomography Time in Patients with Acute Ischemic Stroke Across COVID-19 Periods in the Emergency Department: A Multi-Center Cohort Study

**DOI:** 10.3390/medsci13010031

**Published:** 2025-03-15

**Authors:** Yu-Lin Hsieh, Ching-Fang Tiffany Tzeng, Maha Khan, Andrew Shedd, Thomas Damrow, Dahlia Hassani, Matthew Danley, Jaydeep Shah, Jennifer Walker, Eric H. Chou

**Affiliations:** 1 Department of Medicine, Brigham and Women’s Hospital, Boston, MA 02115, USA; 2 Harvard Medical School, Boston, MA 02115, USA; 3 Department of Emergency Medicine, St Barnabas Hospital, Bronx, NY 10457, USA; 4 Department of Emergency Medicine, Baylor Scott and White All Saints Medical Center, Fort Worth, TX 76104, USA; 5 Anne Marie Burnett School of Medicine, Texas Christian University, Fort Worth, TX 76104, USA; 6 Department of Anesthesiology, Baylor Scott and White All Saints Medical Center, Fort Worth, TX 76104, USA

**Keywords:** door-to-CT time, acute ischemic stroke, sociodemographic, COVID

## Abstract

Introduction: Stroke is the fifth leading cause of death and long-term disability in the United States. The current guideline for stroke management includes a 25 min timeframe from door-to-computed tomography time (DTCT). However, sociodemographic backgrounds may impact the DTCT in acute stroke patients. Methods: This was a retrospective, multicenter, cohort study between January 2018 and August 2022 throughout North Texas. The primary endpoint was DTCT ≤ 25 min upon arrival to hospital for all patients suspected of acute ischemic stroke. Results: During the study period, a total of 23,364 patients were included. Only 4468 patients (19.1%) had DTCT times less than or equal to 25 min, and 16,464 patients (70.5%) had DTCT times more than 25 min. In our cohort, Black (OR 1.35; 95% CI 1.23–1.49) and Asian patients (OR 1.33; 95% CI 1.01–1.74) were more likely to have DTCT > 25 min compared to White patients. Hispanic patients (OR 1.20; 95% CI 1.07–1.34) were more likely to have DTCT > 25 min compared to non-Hispanics. Patients presenting during the COVID (OR 1.45; 95% CI 1.34–1.57) and post-COVID period (OR 1.46; 95% CI 1.30–1.65) were more likely to have DTCT > 25 min compared to the pre-COVID period. Conclusions: We demonstrated a discrepancy in DTCT time for acute ischemic stroke patients based on their race and ethnic population and an increase in DTCT time after the start of COVID-19, which has persisted after the pandemic. These diverse factors highlight the complex interplay of logistical, organizational, and healthcare challenges that have influenced DTCT time.

## 1. Introduction

Stroke is the fifth leading cause of death and long-term disability in the United States, with an estimated prevalence of 7 million individuals affected as of 2019 [1,2]. Studies have shown that earlier administration of intravenous (IV) thrombolytic therapy has been associated with higher rates of favorable clinical outcomes in acute ischemic strokes [3] which is consistent with the Get With The Guidelines-Stroke (GWTG-S) quality improvement program recommendation of IV thrombolytic therapy within 60 min of arrival to the hospital (door-to-needle time, DTNT) [4]. Improved healthcare access and enhanced emergency stroke care protocols have led to a reduction in stroke-related mortality. Brain imaging is required before the administration of IV thrombolytic therapy, and the GWTG-S recommends computed tomography (CT) of the brain within 25 min of arrival to the hospital (door-to-CT time (DTCT) ≤ 25 min) [3], making the achievement of this guideline a precursor to achieving good clinical outcomes. Protocols for stroke management established by the American Heart Association (AHA) and American Stroke Association (ASA) include a 25 min timeframe from door to CT time (DTCT) [5,6]. Minimizing any source of delay in imaging can significantly improve morbidity and mortality for patients experiencing stroke.

In a prior analysis of data from the GWTG-S quality improvement program, only 41.8% of patients with ischemic stroke achieved DTCT ≤ 25 min, with a range of 0% to 100% across hospitals [3]. Since then, a systematic review in 2021 highlighted many factors that are associated with delayed DTCT, including clinical presentation of posterior circulation stroke, presence of nausea or vomiting, and assessment-related delays [7]. Specific factors that have been found to be associated with imaging-related delays include lack of CT scanner availability, airway, or hemodynamic compromise, and waiting for renal function testing [7]. Recent studies focus on the last known well-to-arrival time and neighborhood-level socioeconomic status [8,9]. Despite improved knowledge on barriers to DTCT, there is relatively limited literature assessing the role of sociodemographic factors, and of the literature that exists, there is conflicting evidence regarding the effects of ethnicity, race, sex, age, and disability [3,7,10,11,12,13].

Few studies have examined race/ethnic differences in secondary stroke care metrics, such as DTCT time, despite well-established disparities in acute stroke care [7]. The most recent study regarding this topic was published in 2021 using the Florida Stroke Registry [7]. The results revealed significant disparities in receiving DTCT ≤ 25 that affect female, Black, and non-Hispanic patients who had strokes, despite notable improvements in this acute stroke care metric from 2010 to 2018. To our knowledge, there have been no studies of this kind done in Texas. According to 2021 United States Census Bureau data, Texas has a particularly high percentage of Hispanic or Latino residents (39.7%), compared to only 26.4% in Florida, which may influence the data. Further research into the relationship between sociodemographic factors and achievement of GWTG-S guidelines will help to target specific interventions that will be essential in eliminating racial/ethnic disparities in stroke health care.

Besides different patient demographics, Emergency Departments (ED) faced unprecedented challenges, including increased patient volumes, resource constraints, and rigorous infection control protocols during COVID-19 times. Adapting to these challenges likely increased the DTCT in acute stroke patients. Yoshimoto et al. demonstrated that patients during the pandemic had a 2 min longer median door-to-imaging time [14]. Siegler et al. also showcased that patients treated during COVID-19 were less likely to receive thrombolysis within 60 min of arrival [15]. Kobayashi et al. showed door-to-balloon time for ST elevation myocardial infarction patients was significantly prolonged during the pandemic [16]. A recent systematic review in 2022 highlighted COVID-19 screening processes, the need for chest CT scans, and multidisciplinary consultation as influencing factors for DTCT and intravenous thrombolysis treatment times [7,17].

These diverse factors highlight the complex interplay of logistical, organizational, and healthcare challenges that have influenced DTCT time. However, sociodemographic factors remain insufficiently examined and are crucial to healthcare equality [18]. Identifying disparities can help address inequities and ensure that all patients, regardless of background, receive timely care. Of the literature that addresses barriers to DTCT, there is conflicting evidence regarding the effects of ethnicity, race, sex, age, and disability. Polineni et al. demonstrated that women and Black patients were less likely to achieve DTCT goals [7]. De Havenon et al. showed that increased death rates were associated with social deprivation index, urban location, unemployment rate, and proportion of Black race and Hispanic ethnicity [19]. This retrospective multicohort center study aimed to investigate the association between sociodemographic disparities and DTCT time in patients with acute ischemic stroke (AIS) in North Texas. Additionally, we examined the impact of COVID-19 across different pandemic periods to assess its influence on stroke care disparities.

## 2. Methods

### 2.1. Study Design and Setting

This is a retrospective, multicenter, cohort study conducted from 1 January 2018 to 16 August 2022 in five urban EDs of Baylor Scott & White Healthcare System in North Texas. Potential patient encounters were initially identified through a search of the electronic medical record (EMR) of all study sites by using the International Classification of Disease-10 (ICD-10) codes specific to the primary diagnosis of acute ischemic stroke and transient ischemic attack. The Baylor Scott & White Health Institutional Review Board approved this project (reference ID: 022-169) and exempted the requirement for informed consent due to the retrospective and non-invasive nature of the study. This article followed the Strengthening the Reporting of Observational Studies in Epidemiology (STROBE) guidelines. We adhered to all tenets of the Declaration of Helsinki.

### 2.2. Participants Selection

The study encompassed an urban patient population enrolled in the Baylor Scott & White Health Care System. These EDs were affiliated with both community and teaching hospitals and included trauma center designation levels I through IV located in urban areas. Adult patients (18 years or older) who presented with stroke-like symptoms based on the ED code stroke protocol were included in the study. The suspected diagnosis of acute ischemic stroke was made by the emergency physician. Per AHA/ASA guidelines, stroke is recognized per the “FAST” algorithm. The signs include facial droop, arm weakness, slurred speech, and time of onset [20]. Patients were excluded from the study if they were under the age of 18, arrived at the hospital over 24 h symptom onset, were diagnosed with stroke sub-types other than ischemic, had concomitant traumatic injury, or had significant missing data. A team of research staff reviewed the EMR to ensure eligibility and collect data. Information was ascertained on each patient encounter, including patient demographics, medical history, vital signs, laboratory values, treatment course, and clinical outcomes. If a patient presented more than once during the study period, data from only the first presentation were retained for analysis.

### 2.3. Outcome Measure

The primary endpoint was DTCT ≤ 25 min upon arrival at the hospital for all patients suspected of acute ischemic stroke. We followed guidelines from the US Centers for Disease Control and Prevention to classify patients’ self-identified race and ethnicity into the following categories: Race (White, Black, Asian, and others) and ethnicity (Hispanic vs. non-Hispanic) [7]. The secondary outcome was the association between DTCT and patient age, gender, comorbidities, and COVID period. COVID status was classified as pre-COVID, COVID, and post-COVID. Pre-COVID was defined as any time before March 2020. COVID was defined as anytime between March 2020 and February 2022. Post-COVID was defined as any time after February 2022.

### 2.4. Data Collection

Data for this study were systematically retrieved from the Electronic Medical Records (EMR) system, which was consistently used across all EDs involved in the research. The data collected comprised a wide range of patient-specific information, including demographic characteristics, smoking history, and relevant medical history. Additional details such as vital signs (e.g., blood pressure, heart rate, respiratory rate, and temperature), ED length of stay, laboratory test results, radiology findings, and clinical outcomes were all included in the dataset. The data were extracted from the EHR database by a trained research investigator, who ensured consistency and accuracy across all variables. The collected data were then entered into a secure, encrypted Microsoft Excel spreadsheet (Microsoft Excel 2010; Microsoft Corporation, Seattle, WA, USA) for further processing and analysis. The encrypted spreadsheet was subsequently reviewed and verified by multiple co-investigators of the study team, who conducted thorough checks to ensure the accuracy and completeness of the data before proceeding with the analysis. This multi-step review process helped to minimize errors and guarantee the reliability of the data collected from the diverse range of EDs involved in the study.

### 2.5. Statistical Analysis

In the univariable analysis, continuous variables were presented as means with SD, and categorical variables were presented as frequencies with percentages. The Student’s t-test and the Pearson chi-square test were used to compare the differences for continuous and categorical variables, respectively. Multivariate logistic regression was performed to evaluate the factors associated with delayed DTCT time, including COVID status, race, ethnicity, and sex disparities. A *p*-value < 0.05 was considered statistically significant. All statistical analyses were performed using Stata software (StataCorp. 2023. Stata Statistical Software: Release 18. College Station, TX, USA: StataCorp LLC. software).

The odds ratio (OR) was selected to measure the association between variables and the primary outcome. Multivariable logistic regression analyses were performed to examine the associations between independent variables and outcomes. All available independent variables were considered in the regression model, regardless of whether they were scored as significant by univariate analyses. The stepwise variable selection procedure, with iterations between the forward and backward steps, was applied to obtain the final regression model. Significance levels for entry and to stay were set at 0.15 to avoid exclusion of potential candidate variables. The final regression model was determined by sequentially excluding individual variables with a *p*-value > 0.05 until all regression coefficients were significant.

## 3. Results

### 3.1. Patient Characteristics

**Figure 1:**
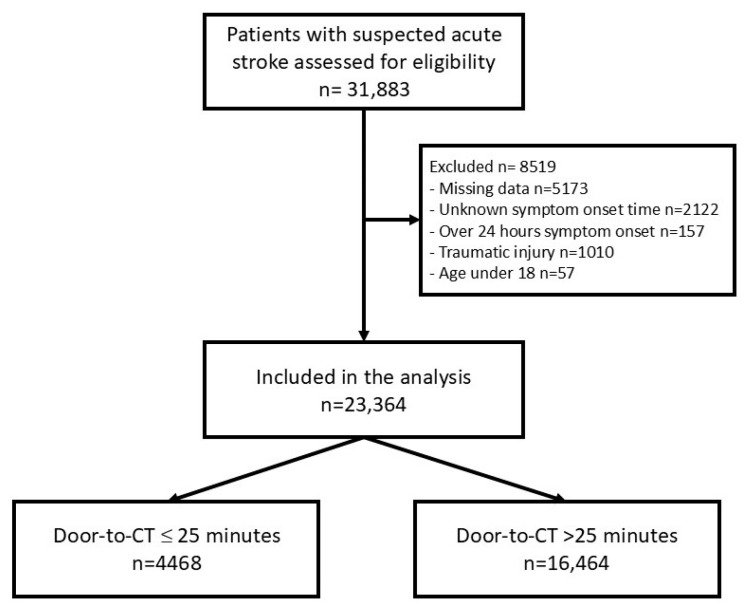
Flow chart of patient selection.

During the study period, a total of 31,883 patient encounters met eligibility criteria. Of these, 23,364 encounters were included in the final analysis (Figure 1). The mean age of the participants was 69 ± 15 years with a nearly even gender distribution, as 49.7% of the participants were male. A majority were White (*n* = 17,345, 74.2%), 20.3% were Black (n = 4745), 1.9% were Asian (n = 454), and 13.5% were Hispanic ethnicity (n = 2807) (Table 1). 4468 patients (19.1%) had DTCT times less than or equal to 25 min, and 16,464 patients (70.5%) had DTCT times more than 25 min. There were significant differences between the two groups in terms of patient age, race/ethnicity, Emergency Severity Index (ESI), and underlying chronic comorbidities, including dementia, chronic renal disease, and chronic liver disease. Patients in the pre-COVID, COVID, and post-COVID phases were 6852 (29.3%), 13,593 (58.2%), and 2919 (12.5%), respectively (Table 1).

### 3.2. Outcome Measures

Multivariable logistic regression analysis is shown in Table 2. Black (OR 1.35; 95% CI 1.23–1.49) and Asian patients (OR 1.33; 95% CI 1.01–1.74) were more likely to have DTCT > 25 min compared to White patients. Hispanic patients (OR 1.20; 95% CI 1.07–1.34) were more likely to have DTCT > 25 min compared to non-Hispanic patients. Patients with chronic liver cirrhosis (OR 1.31; 95% CI 1.16–1.49) and chronic renal disease (OR 1.13; 95% CI 1.04–1.22) were more likely to have DTCT greater than 25 min. Patients evaluated during COVID (OR 1.45; 95% CI 1.34–1.57) and post-COVID (OR 1.46; 95% CI 1.30–1.65) were more likely to have DTCT > 25 min compared to patients evaluated in the pre-COVID period.

## 4. Discussion

This study offers crucial insights into the disparities in acute ischemic stroke care, particularly concerning DTCT times, which directly impact the timely administration of life-saving interventions like tPA and thrombectomy. Our findings underscore a significant racial and ethnic inequality, revealing that Black, Asian, and Hispanic patients experience notably longer door-to-CT times compared to other groups, deviating from the best practice guidelines. The identification of these disparities is vital for healthcare systems aiming to optimize stroke care. Moreover, our study highlights the significant role that the patient race and the post-COVID healthcare environment play in influencing these delays. Our findings suggest that delays in DTCT times have not returned to pre-pandemic levels, indicating that systemic challenges remain even after the acute phase of the pandemic. Moreover, racial and ethnic disparities persist across all time periods, reinforcing the need for equity-focused interventions in stroke care. These results not only call attention to the urgent need for targeted interventions to address these inequities but also offer a roadmap for future research and policy development aimed at improving stroke outcomes across all patient populations.

In our study, Black (OR 1.35; 95% CI 1.23–1.49) and Asian patients (OR 1.33; 95% CI 1.01–1.74) were more likely to have DTCT > 25 min compared to White patients, and Hispanic patients (OR 1.20; 95% CI 1.07–1.34) were more likely to have DTCT > 25 min compared to non-Hispanic patients. Previously, multiple studies have demonstrated similar results [3,7,10,11,12,13,21]; however, others have also reported shorter DTCT times for Hispanic patients. This could be explained through systemic and personal factors. At the system level, implicit bias due to relative lack of diversity [22], socioeconomic status, geographic distance, limited access to health care, and lower rates of arrival by EMT services have been proposed as possible causes. On the personal level, delays in DTCT are often attributed to gaps in medical literacy, family support and connection, delays in recognition of symptoms by patient or medical staff, variation in mode of arrival to the hospital, and difficulties presented using in-person or virtual translation services [7,23,24,25,26]. For example, it can be difficult to rapidly communicate the intricacies of symptom timing and evolution across language barriers [27]. Thus, even though considerable efforts may be put forth by medical staff, critical moments may be lost waiting for an interpreter rephrasing questions to accurately screen for stroke-like symptoms [3,7,21,28,29,30,31,32]. However, further research is needed to elucidate the most impactful causes of these disparities.

DTCT times were also significantly affected by the COVID-19 pandemic. In our study, we found that patients presenting during COVID (OR 1.45; 95% CI 1.34–1.57) and post-COVID (OR 1.46; 95% CI 1.30–1.65) were more likely to have DTCT > 25 min compared to the pre-COVID period. Though stroke protocols did not change during this time, and stroke volume may have decreased [33], it is apparent that the delivery of care before COVID was different than during and post-COVID. However, in our literature review, there has been inconsistency in the results. Most studies found no statistically significant change in DTCT time [33,34,35,36,37,38]. One abstract presented at the International Stroke Conference 2022 showed shortened DTCT by 1 min [39]. Another study found improvements in DTCT times during the pandemic [40]. However, they either failed to achieve statistical significance or lacked peer review. Two other studies reported increased DTCT times. Katsanos et al. studied a different population who received tPA or mechanical thrombectomy [41]. Hu et al. showed a 2 min difference but did not provide the percentage of patients that achieved the 25 min DTCT goal [42]. Our study is the first showing a significant delay in DTCT time using a large sample size after the pandemic.

The COVID pandemic forced EDs to incorporate numerous changes in the delivery of medical care. Changes in workflow, including COVID screening, extra precaution with personal protective equipment, and mandatory masking [43], may contribute to the delay in DTCT we observed. Today the turnover time for COVID screening can be as high, which has dramatically decreased since the beginning of the pandemic [44]. Moreover, the mandatory use of masks helped prevent the spread of airborne pathogens but made communication more challenging. Masking may have hindered the ability of healthcare personnel to accurately triage and examine patients, especially in those presenting with facial droop or slurred speech [45,46,47,48,49]. Other studies suggest increased respiratory, gastrointestinal, or systemic symptom screening by both ED triage and stroke teams, concern over isolation precautions, donning of PPE, and overall increased caution during every phase of patient care were all possible sources of delays [41].

The COVID pandemic also led to increased ED boarding and increased provider burnout [50,51,52,53]. Increased ED crowdedness has been shown to negatively impact DTCT times [54,55]. Additionally, many institutions were short-staffed during the pandemic, and health care workers were working more frequently or for longer hours [56,57,58,59]. Increased ED boarding and rate of burn out may have had a synergistic effect on the increased DTCT time we observed in our study.

## 5. Limitation

There are several limitations to our study. Firstly, due to the retrospective study design and the inability to control for unmeasured confounders, we are limited to reporting associations rather than establishing causality. Unmeasured confounding factors, such as ED crowding, variations in staffing, and differences in hospital protocols, could have influenced DTCT times. Second, the analysis was limited to our healthcare system in North Texas and may have limited external generalizability to other regions. However, our results are consistent with prior studies, including those from large regional datasets, suggesting these disparities are widespread and not due to local practice patterns [7]. We believe it is important to re-emphasize this race/ethnic disparity in CVA management from our cohort. Thirdly, we were not able to identify patients’ mode of arrival to the hospital, the time between symptom onset and arrival, or patients’ spoken language due to the data set presentation in our system based on the limitation of the retrospective design. Fourthly, our data only consists of months after the COVID pandemic. Further effects of the pandemic and recovery from the pandemic cannot be monitored or illustrated with our current data and the retrospective setting. Our study did not include data regarding patient outcomes, so we are unable to assess how these changes in DTCT time affected our patients’ recovery. Lastly, while our study focuses on stroke care disparities rather than incidence rates, we recognize that post-COVID conditions, including increased vascular inflammation and coagulopathy, may contribute to a higher burden of stroke. Future studies are still needed on these topics.

## 6. Conclusions

We demonstrated a discrepancy in DTCT time for acute ischemic stroke patients based on their race and ethnic population. Black and Asian patients were more likely to have DTCT > 25 min compared to White patients, and Hispanic patients were more likely to have DTCT > 25 min compared to non-Hispanic patients. An increase in DTCT time was observed after the start of COVID-19, which has persisted after the pandemic.

## Figures and Tables

**Table 1:** Patient characteristics.

Patient Characteristics	All (n = 23,364)	Door to CT ≤ 25 min (n = 4468)	Door to CT > 25 min (n = 16,464)	*p*-Value
Age (year), mean ± SD	69 ± 15	70 ± 15	69 ± 15	<0.01
Sex, n (%)				0.14
- Male	11,617 (49.7)	2255 (21.8)	8102 (78.2)	
- Female	11,747 (50.3)	2213 (20.9)	8362 (79.1)	
Race/Ethnicity, n (%)				<0.01
- White	17,345 (74.2)	3481 (77.9)	12,161 (73.9)	
- Black	4745 (20.3)	763 (17.1)	3369 (20.5)	
- Asian	454 (1.9)	72 (1.6)	346 (2.1)	
- Other	820 (3.5)	152 (3.4)	588 (3.6)	
- Hispanic	2807 (13.5)	553 (12.5)	2254 (13.8)	
Triage acuity level (Emergency Severity Index)				<0.01
Resuscitation, n (%)	1686 (8.1)	725 (16.4)	961 (5.9)	
Emergent, n (%)	12,604 (60.6)	3361 (76.0)	9243 (56.4)	
Urgent, n (%)	6381 (30.7)	333 (7.5)	6048 (36.9)	
Less Urgent, n (%)	125 (0.6)	2 (0.0)	123 (0.8)	
Non-Urgent, n (%)	3 (0.0)	0 (0.0)	3 (0.0)	
Hypertension, n (%)	19,599 (83.9)	3826 (85.6)	13,782 (83.7)	<0.01
Diabetes mellitus, n (%)	10,082 (43.2)	1826 (40.9)	7202 (43.7)	<0.01
Coronary artery disease, n (%)	7532 (32.2)	1435 (32.1)	5280 (32.1)	0.95
Congestive heart failure, n (%)	6408 (27.4)	1347 (30.1)	5061 (30.7)	0.45
COPD, n (%)	3995 (17.1)	845 (18.9)	3150 (19.1)	0.74
Chronic renal disease, n (%)	6313 (27.0)	1247 (27.9)	5066 (30.8)	<0.01
Liver cirrhosis, n (%)	2360 (10.1)	420 (9.4)	2360 (11.3)	<0.01
Dementia, n (%)	3547 (15.2)	707 (15.8)	2840 (17.2)	0.02
Smoking history, n (%)	8869 (46.4)	1663 (46.1)	6243 (46.1)	0.98
COVID era				<0.01
Pre-COVID	6852 (29.3)	1519 (34.0)	4425 (26.9)	
COVID	13,593 (58.2)	2397 (53.7)	9859 (59.9)	
Post-COVID	2919 (12.5)	552 (12.4)	2180 (13.2)	
COPD, Chronic obstructive pulmonary disease				

**Table 2:** Multivariable logistic regression analysis for risk factors of having a DTCT of greater than 25 min.

	Odds Ratio (95% CI)	*p*-Value
Race		
White	-	-
Black	1.35 (1.23–1.49)	<0.001
Asian	1.33 (1.01–1.74)	0.04
Other	1.09 (0.90–1.33)	0.38
Hispanic	1.20 (1.07–1.34)	0.002
Insurance		
None	-	-
Commercial	1.16 (1.02–1.32)	0.03
Medicare/Medicaid	1.00 (0.87–1.15)	1.00
Temperature	1.06 (1.02–1.10)	0.004
Mean arterial pressure (MAP)	0.99 (0.99–1.00)	<0.001
Glasgow Coma Scale		
3–8	-	-
9–12	0.45 (0.35–0.56)	<0.001
13–15	0.95 (0.78–1.16)	0.59
Liver cirrhosis	1.31 (1.16–1.49)	<0.001
Chronic kidney disease (CKD)	1.13 (1.04–1.22)	0.005
COVID period		
Pre-COVID	-	-
During COVID	1.45 (1.34–1.57)	<0.001
Post-COVID	1.46 (1.34–1.57)	<0.001

## Data Availability

Not publicly available.
